# Subjective Sleep Measures in Children: Self-Report

**DOI:** 10.3389/fped.2017.00022

**Published:** 2017-02-13

**Authors:** Andrea M. Erwin, Lisa Bashore

**Affiliations:** ^1^Texas Christian University Harris College of Nursing and Health Sciences, Fort Worth, TX, USA

**Keywords:** sleep, subjective, self-report, children, measure

## Abstract

The American Academy of Sleep Medicine (AASM) recently published a consensus statement on the recommended number of hours of sleep in infants and children. The AASM expert panel identified seven health categories in children influenced by sleep duration, a component of sleep quality. For optimal health and general function, children require a certain number of hours of sleep each night. Limited data exist to subjectively assess sleep in this population. Practitioners must evaluate overall sleep quality not simply sleep duration. The purpose of this article is to provide a mini-review of the self-report sleep measures used in children. The authors individually completed a review of the literature for this article *via* an independent review followed by collaborative discussion. The subjective measures included in this mini-review have been used in children, but not all measures have reported psychometrics. Several tools included in this mini-review measure subjective sleep in children but with limited reliabilities or only preliminary psychometrics. Accurate measurement of self-reported sleep in children is critical to identify sleep problems in this population and further detect associated health problems. Ongoing studies are warranted to establish reliable and valid measures of self-reported sleep in children to accurately detect health problems associated with poor sleep quality. This mini-review of the literature is an important first step to identify the most reliable subjective sleep measures in children.

## Introduction

The American Academy of Sleep Medicine (AASM) published a consensus statement on the recommended number of hours of sleep in infants and children (1). For optimal health, children require a certain number of hours of sleep each night. However, limited reported resources support clinicians on how to best measure sleep in children and, more importantly, how to improve sleep in this population. Although variability based on genetics, medical, behavioral, and environmental environments exists in the need for sleep in children, health providers need this information to discuss, educate, and promote better sleep practices in children.

Experts who studied and defined sleep and developed sleep promotion approaches are represented in a number of professional organizations, AASM, American Association of Sleep Technologists, and Sleep Research Society, making widespread dissemination of this information a challenge. Easy access to such information is needed to support evidence-based practice and to determine best self-report sleep measures for children and adolescents, especially in cases without a sleep disorder. This information is not well disseminated among the many clinicians who want to accurately measure sleep but cannot spend an inordinate amount of time accessing the most reliable sleep measures in children, but rather need them easily accessible. A gap in the literature exists to describe best measures of self-reported sleep for children and adolescents without sleep disorders. Practitioners must evaluate overall sleep quality not simply sleep duration as recommended by the AASM consensus statement.

Understanding adult sleep characteristics provides a baseline to study clinical conditions that deviate from the expected norm (2). Children with limited number and quality of hours slept may exhibit many potential health problems if they do not receive adequate sleep. This mini-review describes self-report sleep measures in children, sleep components measured, and tool psychometrics when available. Although much information has been previously reported by Spruyt and Gozal (3), this review further examines the tools that measure the construct of sleep in this population. Clinicians who assess sleep in the pediatric populations want to know the tool used is accurately assessing sleep quality without assuming a sleep disorder. The authors provide a list of frequently used terms to define sleep components in Table 1.

**Table 1:** **Sleep definitions**.

Term	Definition
Sleep quality	A complex phenomenon that includes quantitative (i.e., sleep duration, sleep latency, and number of arousals) and subjective (i.e., depth, restfulness) aspects of sleep, which varies between individuals (5)
Sleep latency	The amount of time required to fall asleep once settling down for the night
Sleep quantity	Refers to all aspects of the sleep period including duration and efficiency
Sleep disturbances	Refers to any situation that interferes with sleep including sleep deprivation and sleep disruption (26)
Use of sleep aids	The use of a substance/medication to induce sleep
Daytime functioning	The role of sleep on daily functioning
Excessive daytime sleepiness	A condition in which an individual feels very drowsy during the day and has an urge to fall asleep when he/she should be fully alert and awake
Sleep deprivation	Inadequate amount of sleep due to poor sleep hygiene, lack of consistent bedtime, caregiving, or developmental stages
Sleep disruption	Fragmentation of sleep related to medication, health issues, pregnancy, or sleep disorders
Sleep efficiency	The total amount of time a person slept divided by the total amount of time spent in bed
Sleep hygiene	Practices necessary to maintain quality nighttime sleep and full daytime alertness
Sleep loss	Getting less sleep than needed for optimal functioning
Sleep debt	The difference between the recommended amount of sleep and the actual amount of sleep
Total sleep time	Total amount of sleep in a 24-h period
Sleep apnea	Characterized by pauses in breathing that prevents air from flowing into or out of a sleeping person’s airways

## Methods

The authors accessed several databases to complete the literature review: Cumulative Index to Nursing and Allied Health Literature (CINAHL), PubMed, Embase, Academic Science Complete, Cochrane Library, and the Joanna Briggs Institute. No time period limits were placed and key terms included sleep, child*, *self-report*, and *subjective*, and *measure*. Inclusion criteria were (a) English language, (b) quantitative and qualitative design, (c) subjective measures of sleep using self-report only, and (d) child able to provide self-reported sleep regardless of age. Exclusion criteria included no parental, custodial, teacher, or other proxy report of sleep. Searches using keywords child* and *self-report sleep* yielded over 200 articles; when *measure* was added to the search 64 articles were identified. Following review of the abstracts, only eight met inclusion criteria. The authors identified two literature reviews of measures of self-reported sleep in children and used those reports’ citations to expand the search. Authors also reviewed *STOP, THAT, and One Hundred Other Sleep Scales* (4) in an effort to exhaust the literature. The reasons included tool unav**a**ilability in the English language, abstracts only available for review, and challenges to access certain tools.

## Measures

Subjective assessment of sleep is a critical component of sleep measurement in children because self-reported sleep in children is as valid as objectives measures of sleep; what children report as troublesome to them must be considered when making decisions about their health. Although the authors of the current mini-review agree with the Spruyt and Gozal (3) report on the lack of psychometrics, validation, and misuse of many pediatric sleep measures, the information derived from the self-reported measurement of sleep in children is clinically valuable. The summary of the self-report tools in this mini-review is found in Table 2.

**Table 2:** **Summary of self-report tools used in children**.

Tool, reference	Tool details	Reliability (global score)	Subjects	Use in pediatrics	Weaknesses
Pittsburgh Sleep Quality Index (PSQI), Buysse et al. (5), Carpenter and Andrykowski (6)	19 items Sleep quality (past month) Included both healthy and unhealthy adult populations	Cronbach’s α = 0.83 Healthy and unhealthy adults	Psychometrics in adults *N* = 52 healthy adults *N* = 62 unhealthy adults *N* = 54 depressed adults	**Ertan et al. (7)** *N* = 71 (6–15 years) 44 with monosymptomatic enuresis and 27 healthy controls Relationship between sleep quality and quality of life	No psychometrics reported in children or adolescents
				**Duarte et al. (8)** *N* = 2,094 (16–23 years) Sleep–wake patterns in Portuguese healthy adolescents	

Epworth Sleepiness Scale, Johns (9), Johns (10), Baumgartel et al. (11)	8 items Sleep propensity (typical behavior) Included both healthy and unhealthy adult populations	Cronbach’s α = 0.73 (healthy adults) Cronbach’s α = 0.88 (unhealthy adults) Cronbach’s α = 0.751 (pregnant women)	Psychometrics in Adults *N* = 87 healthy adults *N* = 54 unhealthy adults *N* = 168 pregnant women	**Duarte et al. (8)** *N* = 2,094 (16–23 years) Sleep–wake patterns in Portuguese healthy adolescents	No psychometrics reported in children or adolescents
				**Melendres et al. (12)** *N* = 180 (2–17 years) 108 with suspected sleep-disordered breathing (SDB) and 72 healthy controls Relationship between SDB and hyperactivity	
				**Moore et al. (13)** *N* = 247 (13–16 years) Relationship between psychological functioning and sleep	

Children’s report of sleep patterns, Meltzer et al. (14)	60 items Sleep patterns, sleep hygiene, and sleep disturbances (most questions reflect typical week) Included healthy and unhealthy children populations	Cronbach’s α ≥ 0.70 (parasomnia scale: Cronbach’s α = 0.64)	*N* = 456 (8–12 years) Healthy and unhealthy children	Preliminary psychometrics of self-report tool development	Only preliminary-reported psychometrics in children
				**Validity** Significant differences between groups (healthy versus unhealthy; age; nappers versus non-nappers) Significant associations between daytime sleepers and associated tool items	

Adolescent Sleep–Wake Scale (ASWS), LeBourgeois et al. (15),	**LeBourgeois et al. (15)** 29 items Sleep quality (past month) Included healthy adolescent populations	**LeBourgeois et al. (15)** Cronbach’s α = 0.80 Italian adolescents Cronbach’s α = 0.86 American adolescents	**LeBourgeois et al. (15)** *N* = 1,348 (12–17 years) 776 Italian and 572 American adolescents Healthy adolescents	**LeBourgeois et al. (15)** Relationship between (1) sleep-hygiene practices and sleep quality and (2) relationship between culture and sleep quality	**LeBourgeois et al. (15)** No psychometrics reported
**Essner et al. (16)**	**Essner et al. (16)** 10 items Included healthy adolescents and adolescents with health conditions	**Essner et al. (16)** Cronbach’s α = 0.81	**Essner et al. (16)** *N* = 491 (12–18 years) Healthy adolescents and adolescents with health conditions	**Essner et al. (16)** Examine factor structure of the ASWS	**Essner et al. (16)** Only preliminary-reported psychometrics in adolescents

Pediatric Daytime Sleepiness Scale, Drake et al. (17)	8 items Daytime sleepiness (typical behavior) Included healthy children population	Cronbach’s α = 0.80 Healthy children	*N* = 442 students (11–15 years) Healthy children	Relationship between daytime sleepiness and academic outcomes	Did not report item-analyses, non-response analyses, or standardization/norms development

Sleep Disturbances Scale for Children, Bruni et al. (18)	26 items Sleep disorders (previous 6 months) Included healthy and unhealthy children populations	Cronbach’s α = 0.71 healthy and unhealthy children Cronbach’s α = 0.79 healthy children Cronbach’s α = 0.71 sleep disorder group	*N* = 1,304 (6.5–15.3 years) Maternal report Factor analysis yielded six factors Validity supported in discriminating healthy and unhealthy children	**Vilela et al. (19)** *N* = 531 (10–18 years) Assessed excessive sleepiness and associated factors	**Bruni et al. (18)** Maternal report
					**Vilela et al. (19)** Cross-sectional design

Cleveland adolescent sleepiness questionnaire, Spilsbury et al. (20)	16 items (15 items removed following factor analysis) Excessive daytime sleepiness in adolescents (past month) Included both healthy adolescents and adolescents with SDB	Cronbach’s α = 0.89 Healthy adolescents	Psychometrics in children *N* = 473 (11–17 years) 411 healthy adolescents and 62 adolescents with SDB	Self-report tool development	Did not report standardization/norms development

Sleep self-report, Owens et al. (21)	23 items (3 items removed following factor analysis) Sleep behavior (past week) Included healthy children population	Cronbach’s α = 0.88 Healthy children	Psychometrics in Children *N* = 691 (6–11 years) School-based study healthy children	Examined common sleep behaviors in elementary students (6–11 years) Survey designed for 7–12 years	Limited to medically focused sleep disturbances

School sleep habits survey, Wolfson and Carskadon (22)	140 items Sleeping–waking behaviors (past 2 weeks) Included healthy adolescent population	Cronbach’s α = 0.66–0.77 in five scales Healthy adolescents	*N* = 3,120 (13–19 years) Validated in adolescents	Relationship between sleep/wake habits and daytime sleepiness, high school grades, depressed mood, and other daytime concerns affecting sleep in adolescents	Only preliminary-reported psychometrics in children-adolescents

### Pittsburgh Sleep Quality Index (PSQI)

The PSQI was developed to measure the complex multidimensional phenomenon of sleep quality and address the components of what constitutes sleep including: sleep quality, sleep latency, sleep duration, habitual sleep efficiency, sleep disturbances, use of sleep medications, and daytime dysfunction in the past month (5). The PSQI is composed of 19 self-rated questions and five questions rated by a bed partner or roommate. Only the self-rated items are used in scoring the scale. The dimension scores are summed to produce a global score (range 0–21). Cronbach’s alpha was 0.83 for the global score following examination in a group of healthy and unhealthy adults. Test–retest reliability (average interval of 28 days) assessed with a subset of 91 patients determined the internal consistency was 0.85 for the global score and 0.65–0.84 for the subscales (6).

The PSQI was used in adolescents and children to examine sleep disturbances and the association with health problems (7) and school performance (8). Ertan et al. (7) explored the relationship between sleep and quality of life in 44 children with monosymptomatic enuresis. Duarte et al. (8) conducted a correlational epidemiological, cross-sectional study of 2,094 students aged 16–23 years (*M* = 15.82 ± 1.25) in regards to their sleep patterns and academic performance. Neither study established the psychometrics for the PSQI limiting the usefulness of the PSQI in pediatrics.

### Epworth Sleepiness Scale (ESS)

The ESS measures daytime sleepiness and consists of eight items (situations) where individuals assess how likely they would fall asleep (9). A sum of responses is calculated for a total score ranging from 0 to 24 with higher numbers representing greater sleepiness (9). The original intent was for subjects to use the recent past as a frame of reference in completing the scale and for clinicians and researchers to instruct subjects to focus on dozing and not tiredness or sleepiness.

Psychometrics were initially conducted by Johns (10) comparing adult students with sleep disorders and a normative sample of medical students. Cronbach’s alpha was 0.88 in adults with sleep disorders and 0.73 in medical students, respectively (10). Further psychometrics were performed and confirmatory analysis done in one half of a sample of pregnant women (*N* = 168) (11). The overall Cronbach’s alpha of the ESS was acceptable reliability of an established instrument (α = 0.751). The Cronbach’s alpha for the overall score without item seven (α = 0.708) was less than when it was included in the overall score (α = 0.751) (10).

The ESS has been used in children and adolescents to examine the relationship of sleep-disordered breathing and hyperactivity (12) and psychological functioning and sleep (13). However, no psychometrics for the ESS in children has been established. Melendres et al. (12) were the first to use a modified ESS in children. However, their study included both parent- and child-reported sleepiness. They did not report how many children provided self-reported daytime sleepiness and, again, did not perform psychometrics. Moore et al. (13) used the terms sleep duration, any variation in sleep duration, and sleepiness as a proxy for sleep disorders.

### Children’s Report of Sleep Patterns (CRSPs)

The CRSP was developed and tested in 456 children aged 8–12 years to measure sleep patterns in children (14). The Cronbach’s α is 0.70–0.76 for the sleep disturbances scales (one of the three modules contained within the CRSP). The authors did not provide reliabilities for the other subscales; sleep pattern and sleep hygiene indices. The CRSP was validated through multimodal efforts using parental report and child report, and the objective measure of actigraphy.

### Adolescent Sleep–Wake Scale (ASWS)

The ASWS has been used to examine sleep quality (15). The ASWS measures the quality of sleep based on specific nighttime behaviors including going to bed and falling asleep (latency), maintaining sleep, reinitiating sleep, and returning to wakefulness. The ASWS is a 29-item self-report measure that assesses sleep quality in 12- to 18-year-old adolescents in the past month (15). Reported Cronbach α was between 0.80 and 0.86 in this population.

The factor structure of the ASWS was examined in a recent study by Essner et al. (16) in a population of adolescents with and without health conditions. Following factor analysis, the total scale was reduced to 10 items. Falling asleep and reinitiating sleep were combined into one factor loading in the final analysis. Reported Cronbach α was 0.81 for the total sample.

### Pediatric Daytime Sleepiness Scale (PDSS)

The PDSS was developed and tested in a population of 442 middle school students (11–15 years) to examine the relationship of daytime sleepiness and academic outcomes (17). Psychometrics of the PDSS included a total of eight items with acceptable factor loadings. Internal consistency (Cronbach’s alpha) for the total scale was 0.81 and 0.80 on the split-half sample (17).

The PDSS was designed to measure daytime sleepiness. The researchers indicated this measure assesses daily sleep patterns including total sleep time, weekday bed time, and weekday wake time (17). The recall period that the child was asked to respond to the items is unclear.

### Sleep Disturbances Scale for Children (SDSC)

The SDSC is a 27-item scale tested in 1,304 children and adolescents ages ranging from 6.5 to 15.3 years in Rome to evaluate sleep disturbances in children (18). However, Bruni et al. (18) had mothers to complete the measures rather than self-report. The SDSC had good internal consistency between the control and sleep disorders groups; Cronbach’s alpha was 0.79 and 0.71, respectively, and Cronbach’s alpha on the total score was (*r* = 0.71) (18).

The SDSC was further evaluated in a population of 531 adolescents in both public and private schools to measure factors that influence excessive daytime sleepiness in this population (18). Vilela et al. (19) included items on sleep deficit on the weekends compared to the weekdays to determine a sleep deficit score. This study was a cross-sectional design and may require more longitudinal assessment (19).

### Cleveland Adolescent Sleepiness Questionnaire (CASQ)

The CASQ was developed specifically to measure daytime sleepiness in adolescents. The CASQ was used in adolescents with sleep-ordered breathing and a healthy adolescent sample and was developed to measure sleepiness in adolescents 11–17 years of age (20). Exploratory factor analysis (EFA) was first conducted on half of the healthy sample (*N* = 181) and revealed a final 4-factor solution explaining 55% of the variance; “sleep in school,” “alert in school,” “sleep in the evening,” and “sleep during transport.” Psychometrics indicated a Cronbach’s alpha 0.89. Whether this tool is useful in a healthy adolescent population to clinically assess sleep is not clear.

### Sleep Self-Report (SSR)

The SSR is a 26-item measure developed and administered to 691 grade school children aged 7–12 years to measure sleep habits and prevalence of common medical and behavioral sleep disturbances in young children (21) and was designed to assess components similar to the parent version of this tool, the Children’s Sleep Habits Questionnaire. Components include bedtime behavior, sleep onset, sleep duration, behavior occurring during sleep, and night wakenings, parasomnias, and morning waking/daytime sleepiness. Factor analysis yielded 23 items. A total score resulted in an alpha coefficient of.88 (21).

The authors propose that this tool measures sleep habits and medical/behavioral disturbances. The tool is limited to medically focused sleep disturbances, such as pain and possibly night terrors, and is not comprehensive.

### School Sleep Habits Survey

The goal of the study was to document the relationship of sleep/wake habits and daytime sleepiness, high school grades, depressed mood, and other daytime concerns affecting sleep in adolescents (22). The survey includes items on school performance (grades), daytime sleepiness, sleep/wake problems, and depressive mood. This tool has multiple items on trauma or potential traumatic injury that do not have adequate rationale for their inclusion in this tool. Further, the authors do not discuss these items within their narrative when reporting outcomes of sleep in adolescents (22).

The scoring of this tool may not be apparent to clinicians who want to use this survey in practice and is burdensome to the general clinician for use in clinical practice to make assumptions about sleep quality in normal adolescence. The items that were labeled were found on the Sleep for Science webpage (23).

## Discussion

Measuring all components of sleep quality in children is important due to the potential health problems sequential to poor sleep quality. Subjective measures rely on having the appropriate developmental tool available to the population of interest. Measurement results may vary based on ability of children to provide an accurate self-report. Many tools lack reported psychometric evaluation and have limited reliability.

The self-report measures in this mini-review were chosen based on the authors’ access to the measures and that the tool was used to measure self-reported sleep in children and adolescents. Many of the reviewed subjective measures lacked consistent use of terminology in regards to sleep components or lack of rationale for chosen components or factors associated with poor sleep (13, 20). Descriptive literature often lacked ongoing large clinical trials to examine tool utility in measuring sleep quality in children over time (5, 9, 14–18, 20–22). When measuring sleep in children with potential limited recall and the potential for chronic poor sleep in children, longitudinal assessment of sleep in children is warranted.

The PSQI and ESS have been used widely in adults with acceptable reliabilities (5, 6, 9–11). Despite the lack of psychometrics of these tools in children and adolescents, the PSQI and ESS have been examined in children and adolescents with sleep disturbances and the association with health problems including academic performance (8), enuresis (7), hyperactivity (12), and psychological functioning (13).

The CRSP (14), The ASWS (16), The PDSS (17), SDSC (18), CASQs (20), and SSR (21) have been developed for children and adolescents and have preliminary psychometrics performed with acceptable reliabilities in the population they were tested.

The SDSC and the CASQ were tested in populations of children with and without sleep disorders and have not been further reported in the literature (18, 20). In addition, the SSR examined sleep habits in young children and the presence of sleep disturbances (21). While the SSR was examined in a normal population of elementary school children, it was designed to identify sleep disturbances. The CASQ was developed to measure daytime sleepiness in adolescents, a typical problem in this population and showed promise in being able to discern between daytime sleepiness and sleep disorders (20). The CASQ when used in another study by Vilela et al. (19) reported that adolescents with higher scores on the CASQ were associated with the presence of sleep disorders. The SDSC also measures sleep disturbances in children with known or presumed sleep disorders. In addition, the SSR was used to examine the relationship between sleep habits and common medical and behavioral sleep disturbances. The CASQ, SDSC, and the SSR have not been repeated in the literature and have only been used to identify children and adolescents with sleep disorders and not a normative sample. Therefore, these tools may not be applicable in assessing sleep quality in healthy children and adolescents in the clinical setting.

The ASWS has been used to measure sleep quality in adolescents (15, 16). The researchers reported the reliabilities of these measures, but the value in assessing sleep in healthy children has not been established over time. Further exploration of these measures in healthy adolescents is needed to address the various reasons why adolescents may have poor sleep quality.

The School Sleep Habits Survey was a lengthy survey used in adolescents to identify the many issues precluding this population from getting adequate sleep. The researchers did not provide rationales for item inclusion. The items, specifically on trauma or injury and their relationship to sleep in adolescents, are not clear.

The AASM consensus statement clearly recommends that health professionals take a proactive approach in addressing, educating and promoting healthy sleep practices in children (1). Children and adolescents have many reasons for poor sleep quality and sleep is critical to everyday functioning and overall health. Addressing sleep quality in all children is necessary. Poor sleep quality impacts academic performance attention, cognition, and mood in adolescents specifically (8, 24). Further, sleep problems in adolescent survivors of cancer have been found to correlate with poorer psychosocial outcomes including depressive symptoms in this population (25).

Sleep is important for overall functioning in healthy children and adolescents. These measures provide support for their use in clinical and research settings. Moore et al. (13) highlight the need to understand the relationship between sleep and psychological functioning, such as depression, in adolescents. Specific targeted questions to children able to provide self-report on their own sleep as they can with the assessment with pain is critical to meeting their entire health care needs.

## Implications

Establishing the psychometrics of these tools is critical to have reliable measures of self-reported sleep in children and adolescents. Many of the sleep measures have not been used over time in the population in which they were initially tested limiting the ability to determine their clinical value and feasibility in clinical practice. Finally, clinicians need sleep measures they can easily administer during a busy clinical day. Ascertaining self-reported sleep in this population yields the most valuable data in the absence of objective sleep measures.

The concept of sleep and the components of sleep important to daily functioning and overall health in children remains unclear to the general clinician. Children reporting sleepiness during the daytime are not alert, able to function well enough to learn, engage in social relationships, engage in athletics, or drive.

One possible explanation for the lack of comprehensive and well-designed subjective measures of sleep is the lack of understanding of the concept of sleep. The authors of this review paper were not able to identify any literature addressing the concept of sleep. Before best measures of self-reported sleep in children can be developed, the concept of sleep requires further attention in healthy children.

## Author Contributions

AE and LB contributed equally to the literature search, summation of the literature, and revisions of the manuscript. All the authors read and approved the final version of this revised manuscript.

## Conflict of Interest Statement

The authors declare that the research was conducted in the absence of any commercial or financial relationships that could be construed as a potential conflict of interest.
